# First report of *Meloidogyne javanica* on Ginger and Turmeric in the United States

**DOI:** 10.21307/jofnem-2019-006

**Published:** 2019-04-26

**Authors:** Abolfazl Hajihassani, Weimin Ye, Brooke B. Hampton

**Affiliations:** 1 Department of Plant Pathology, University of Georgia, Tifton, GA, 31794; 2 Nematode Assay Section, Agronomic Division, North Carolina Department of Agriculture & Consumer Services, Raleigh, NC, 27607; 3 University of Georgia Cooperative Extension Office, Alamo, GA, 30411

**Keywords:** Georgia, Organic vegetables, Pathogenicity, Root-knot nematode, Rhizomes

## Abstract

Ginger (*Zingiber officinale* L.) and turmeric (*Curcuma longa* L.) are two her baceous perennial plant species with rhizomes that are commonly used for flavoring or medicinal purposes. In January 2018, stunting and poorly developed root systems typically associated with plant-parasitic nematode infection were observed on organically grown edible ginger and turmeric in a hoop house in Wheeler County, Georgia. Examination of soil and root samples from symptomatic plants revealed the presence of high populations of root-knot nematodes (*Meloidogyne* spp.). The second-stage juveniles (J2s) were extracted from soil samples as described by Jenkins (1964). Nematode counts were 285 and 155 J2s per 100 cm^3^ soil in the areas planted with ginger and turmeric, respectively. Nematode eggs were recovered from infected root systems using the bleach (1%) and blending method (Hussey and Barker, 1973). Examination of the root samples showed the presence of 840 and 320 eggs per g of roots in ginger and turmeric, respectively. Primary diagnosis of the *Meloidogyne* specimens was done by comparing morphological features observed in the J2s (n = 10) and perineal pattern of females (n = 11) based on the description given by Eisenback and Triantaphyllou (1991) and were tentatively identified as *M. javanica* (Treub, 1885; Chitwood, 1949). For species identification, DNA sequencing was performed using multiple markers located in 18S ribosomal RNA and 5.8S internal transcribed spacer 1 regions, (18S + ITS) (GenBank Accession No. MK390613), 28S domain 2 and 3 (28S D2/D3) (MK385596), cytochrome oxidase subunit I (COI) (MK391558), and subunit II and 16S (COII + 16S) (MK391557) of mitochondrial DNA following methods as described in Ye et al. (2015). PCR assays by species-specific primers were also conducted to confirm species identity as described by Zijlstra et al. (2000).

The blast search results of DNA sequences of 18S + ITS, 28S (D2/D3), COI and COII + 16S revealed the best match as *M. javanica*, *M. incognita* (Kofoid and White, 1912; Chitwood, 1949) and *M. arenaria* (Neal, 1889; Chitwood, 1949) with 99–100% identity. These genes are highly conserved across these three most common root-knot nematode species. However, results of PCR assays by species-specific primers were only positive for *M. javanica* using primers Fjav/Rjav, but negative for *M. incognita* by Finc/Rinc and *M. arenaria* by Far/Rar as described by Zijlstra et al. (2000). Based on morphological characteristics and molecular analyses, the root-knot nematodes infecting ginger and turmeric were identified as *M. javanica*. After confirmation of the nematode species, a test was conducted in the greenhouse to assess the pathogenicity of the nematode on ginger and turmeric. Five seedlings per plant species (cultivars unknown) were grown in 15 cm-diam plastic pots containing equal parts of pasteurized field soil and sand, and then inoculated with 2,000 eggs of *M. javanica*. The egg suspension was added into three holes around the base of each seedling. Non-inoculated seedlings (n2 = 25) were used as controls. Plants were arranged in completely randomized design and grown at 25 ± 3 °C for 10 weeks. At the termination of the experiment, small galls were noticed on the roots of the inoculated seedlings of both ginger and turmeric. No galls were observed on the roots of non-inoculated plants. Egg were extracted from the galled roots (Hussey and Barker, 1973) yielding an average of 1040 ± 96 and 732 ± 54 eggs per g of root of ginger and turmeric, respectively. On ginger, the nematode produced large numbers of galls and egg masses on both primary and secondary (feeder) roots, but the galls produced on turmeric were often observed only on primary roots (Fig. 1). No symptoms of root-knot nematode infestation including galls or water-soaked lesions were observed on the outer surface of rhizomes of both ginger and turmeric. However, the size of rhizomes in the *M. javanica*-infested areas was visibly smaller than that in non-infested areas (Fig. 2). A similar reduction in the growth of turmeric rhizomes was also observed. *Meloidogyne incognita* has been commonly reported as a nematode pest of ginger (Myers et al., 2017) and turmeric (Hall et al., 2017) in the USA and both *M. incognita* and *M. javanica* are known to cause damage on these plant hosts (Ray et al., 1995; Singh and Gupta, 2011). To the best of our knowledge, this is the first report of *M. javanica* on ginger and turmeric in the USA.

**Fig. 1:**
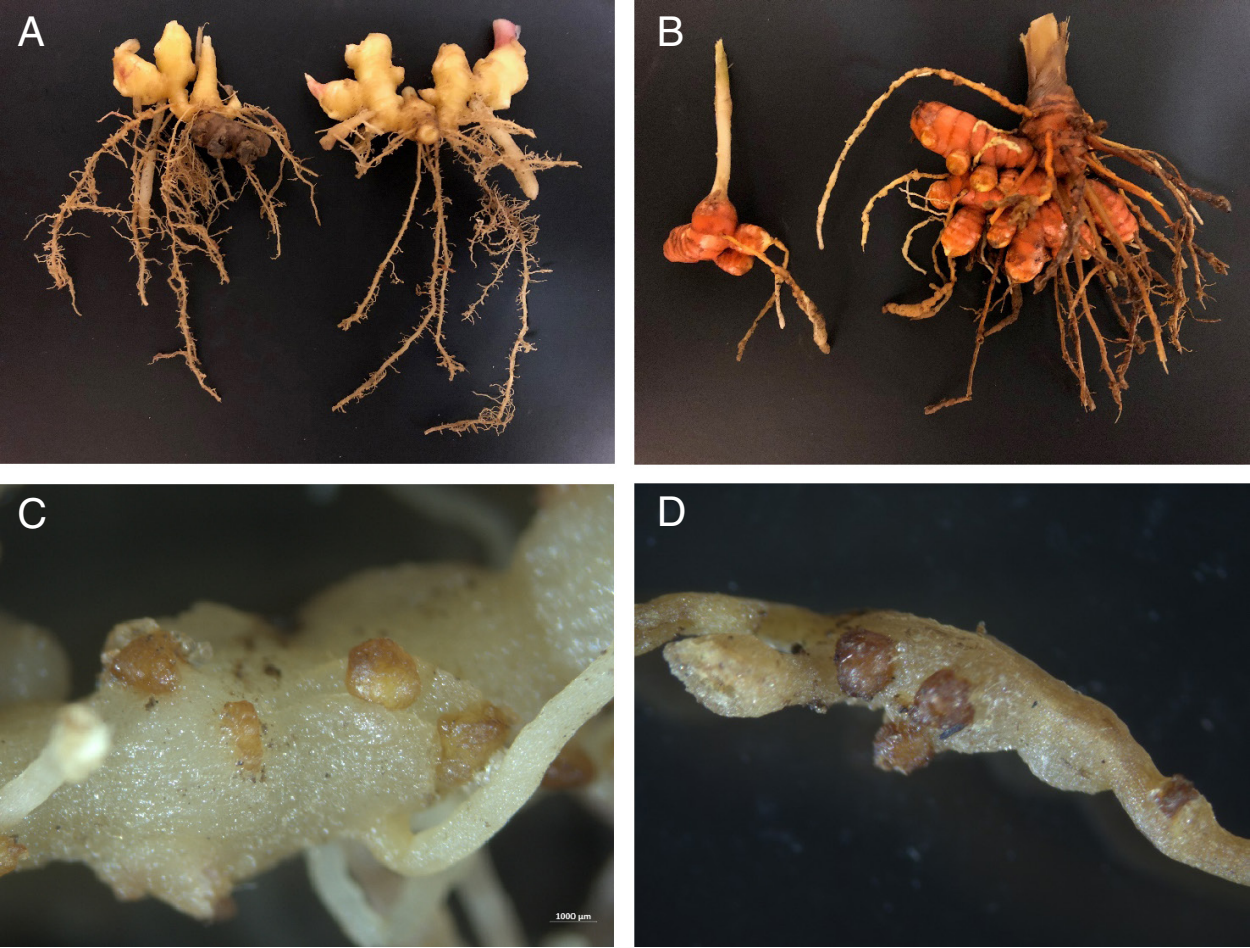
Ginger (*Zingiber officinale*) (A) and turmeric (*Curcuma longa*) infected with *Meloidogyne javanica* from an organic farm in Wheeler County, Georgia showing severely galled roots. Numerous egg masses of the nematode are evident protruding from galled roots of both ginger (C) and turmeric (D).

**Fig. 2:**
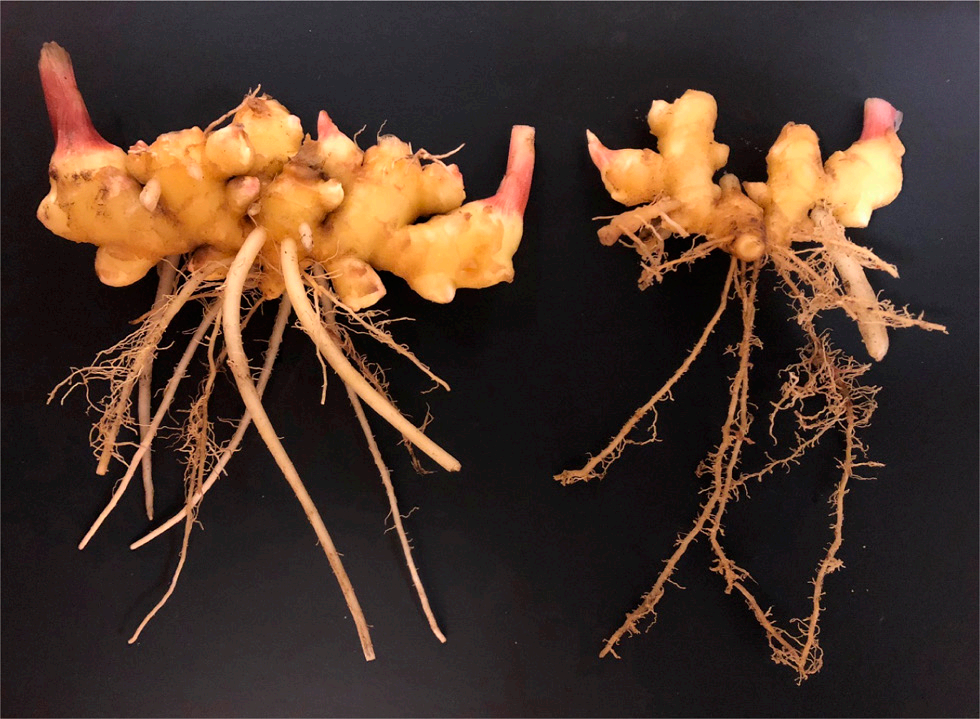
Healthy ginger (left) and ginger infected with *Meloidogyne javanica* (right) showing stunted growth of the rhizomes, collected from nematode-infested soils on an organic farm in Wheeler County, Georgia.
